# Crotalus in Bubonic Plague

**Published:** 1899-11

**Authors:** 


### CROTALUS IN BUBONIC PLAGUE.

The Indians inhabiting the country of the tributaries of the head waters of the Amazon, are subject to a disease, never epidemic but endemic, which resembles in a very marked degree some of the distinctive features of the bubonic plague. There is the same bubonic abscesses, sloughing ulcers, rapid wasting of flesh and speedy failure of the vital forces. The country is covered with dense forests, often impenetrable to the sun, the land low and swampy, and the air full of noxious vapors. The huts are filthy, the food, mostly fish, and the habits of life but little above the animal. The disease, of a bubonic nature, but having no name, is often controlled by a scarification on the skin with the fang of the crotalus, through which is introduced into the circulation of the blood a minute portion of the poison. The poison of the crotalus overpowers that which is raging in the blood. Following this injection of the poison a decoction is given of the cedron, a nut resembling in its medicinal action cinchona, to neutralize the action of any excess of the crotalus and in the destruction of the poison germs of the disease.—*Medical Times*, Sept., 1899.

